# BMP6 Regulates Proliferation and Apoptosis of Human Sertoli Cells Via Smad2/3 and Cyclin D1 Pathway and DACH1 and TFAP2A Activation

**DOI:** 10.1038/srep45298

**Published:** 2017-04-07

**Authors:** Hong Wang, Qingqing Yuan, Min Sun, Minghui Niu, Liping Wen, Hongyong Fu, Fan Zhou, Zheng Chen, Chencheng Yao, Jingmei Hou, Ruinan Shen, Qisheng Lin, Wenjie Liu, Ruobing Jia, Zheng Li, Zuping He

**Affiliations:** 1 State Key Laboratory of Oncogenes and Related Genes, Renji- Med X Clinical Stem Cell Research Center, Ren Ji Hospital, School of Medicine, Shanghai Jiao Tong University, Shanghai 200127, China; 2 Department of Andrology, Urologic Medical Center, Shanghai General Hospital, Shanghai Jiao Tong University, 100 Haining Road, Shanghai 200080, China; 3 Shanghai Institute of Andrology, Ren Ji Hospital, School of Medicine, Shanghai Jiao Tong University, 145 Shangdong Road, Shanghai 200001, China; 4 Shanghai Key Laboratory of Assisted Reproduction and Reproductive Genetics, Shanghai 200127, China; 5 Shanghai Key Laboratory of Reproductive Medicine, Shanghai 200025, China

## Abstract

Sertoli cells are essential for regulating normal spermatogenesis. However, the mechanisms underlying human Sertoli cell development remain largely elusive. Here we examined the function and signaling pathways of BMP6 in regulating human Sertoli cells. RT-PCR, immunocytochemistry and Western blots revealed that BMP6 and its multiple receptors were expressed in human Sertoli cells. CCK-8 and EDU assays showed that BMP6 promoted the proliferation of Sertoli cells. Conversely, BMP6 siRNAs inhibited the division of these cells. Annexin V/PI assay indicated that BMP6 reduced the apoptosis in human Sertoli cells, whereas BMP6 knockdown assumed reverse effects. BMP6 enhanced the expression levels of ZO1, SCF, GDNF and AR in human Sertoli cells, and ELISA assay showed an increase of SCF by BMP6 and a reduction by BMP6 siRNAs. Notably, Smad2/3 phosphorylation and cyclin D1 were enhanced by BMP6 and decreased by BMP6 siRNAs in human Sertoli cells. The levels of DACH1 and TFAP2A were increased by BMP6 and reduced by BMP6 siRNAs, and the growth of human Sertoli cells was inhibited by these siRNAs. Collectively, these results suggest that BMP6 regulates the proliferation and apoptosis of human Sertoli cells via activating the Smad2/3/cyclin D1 and DACH1 and TFAP2A pathway.

Infertility is one of the most serious diseases affecting 10%–15% of the couples worldwide, and half of them are due to male factors. Azoospermia has been defined as the complete absence of sperm in the ejaculate, and it comprises approximately 15% of male infertility^1^,^2^. Spermatogenesis is a complex and elaborated process regulated by the testicular microenvironment or niche, which is composed of Sertoli cells, Leydig cells, blood vessels, growth factors, and cytokines^3^. As the unique somatic cells within the seminiferous tubules, Sertoli cells play essential roles in regulating normal spermatogenesis. The number of Sertoli cells determines the output of male germ cells, because each Sertoli cell supports the fixed number of germ cells^4^, although the capacity in different species is variant^3^. In contrast, the immaturity of Sertoli cells leads to dyszoospermia which is defined as the imperfect formation of spermatozoa. Therefore, it is essential to explore the mechanisms underlying the proliferation, apoptosis and maturity of Sertoli cells, which contributes to uncover the etiology of dyszoospermia and treat human male infertility.

Bone morphogenetic proteins (BMPs) belong to the member of the transforming growth factor-β (TGF-β) superfamily, which activates Smad phosphorylation via binding type I and type II receptors and regulates downstream gene expression. Currently, more than 20 members of TGF-β superfamily have been identified in humans with various functions from osteogenic to nonosteogenic developmental processes, embryogenesis, hematopoiesis, skeletal formation and neurogenesis^5^. BMPs are generally classified as numerous subgroups in terms of their sequence similarity and functions, namely BMP2/4, BMP5/6/7/8a/8b, BMP9/10, and BMP12/13/14/15^6^,^7^. We have recently demonstrated that BMP4 stimulates the division of human Sertoli cells via the Smad1/5 pathway^8^. BMP15 is expressed in the testes and it is specifically localized in rat gonocytes and pachytene spermatocytes^9^. BMP6 plays essential roles in mediating the self-renewal and differentiation of various kinds of stem cells, as evidenced by the following facts: i) BMP6 is required for the differentiation from mesenchymal stem cells to cartilage both *in vivo* and *in vitro*^10^; ii) overexpression of BMP6 promotes the proliferation of germline stem cells (GSCs), and conversely, deficiency of BMP6 leads to the loss of GSCs or germ cells^11^,^12^. BMP6 prevents the differentiation of GSCs by activating Smad1/5 pathway^13^; iii) BMP6 regulates the number of hematopoietic stem cells (HSCs) via combination with BMPR1 receptor^14^, since the production of BMP6 is upregulated by erythropoietin in HSCs to form bone^15^; and iv) recent studies imply that BMP6 regulates neural stem cell (NSC) fate determinations in a time-dependent manner. BMP6 probably promotes the proliferation or differentiation of NSCs in the process of the neurogenesis, and it induces NSC differentiation into astrocytes rather than self-renewal^16^,^17^. In addition, the deficiency of BMP6 may cause cancer, heritable and metabolic disorders, and cardiovascular disease^18^,^19^,^20^. However, the expression, function and mechanisms of BMP6 in regulating the fate determinations of human Sertoli cells remain elusive.

In the current study, we have explored the expression of BMP6 and its receptors as well as the function and signaling pathways of BMP6 in controlling human Sertoli cells. We revealed that BMP6 and its multiple receptors were present in human Sertoli cells, suggesting that BMP6 acts via an autocrine pathway in human Sertoli cells. Functional assays indicated that BMP6 stimulated the proliferation and DNA synthesis of Sertoli cells but inhibited their apoptosis. Meanwhile, BMP6 improved the secretion of SCF and GDNF in human Sertoli cells. Significantly, BMP6 activated Smad2/3 and cyclin D1 pathway and levels of transcription factors DACH1 and TFAP2A. This study provides a novel insight into the molecular mechanisms regulating proliferation and apoptosis of human Sertoli cells.

## Results

### Isolation and Identification of Human Primary Sertoli Cells

Human Sertoli cells were isolated and purified from 32 obstructive azoospermia (OA) patients using a two-step enzymatic digestion and followed by the differential plating as previously described^8^. The viability of freshly isolated cells was over 96%, as measured by trypan blue exclusion assay (data not shown).

**Figure 1:**
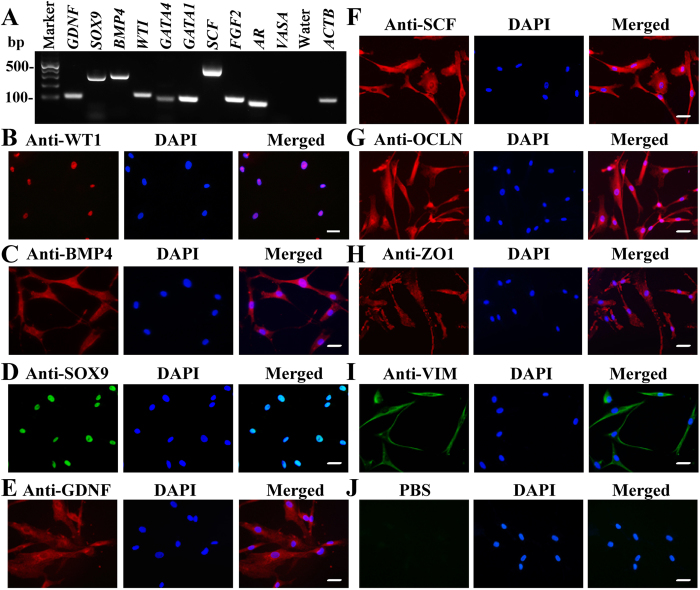
Isolation and identification of human Sertoli cells from OA patients. (**A**) RT-PCR showed the transcripts of *GDNF, SOX9, BMP4, WT1, GATA4, GATA1, SCF, FGF2, AR* and *VASA* in the isolated cells. PCR with water but without cDNA served as a negative control. (**B–I**) Immunocytochemistry demonstrated the protein expression of WT1 (**B**), BMP4 (**C**), SOX9 (**D**), GDNF (**E**), SCF (**F**), OCLN (**G**), ZO1 (**H**), and VIM (**I**) in the isolated cells. Replacement of primary antibodies with PBS was used as a negative control (**J**). The cell nuclei were counterstained with DAPI. Scale bars in (**B–J**) = 20 μm. The data shown in (**A–J**) were representatives from three independent experiments of five patients’ tissues mixed in each experiment.

The isolated human cells from OA patients were identified by a number of various markers for Sertoli cells at both transcriptional and translational levels. RT-PCR showed that *GDNF, SOX9, BMP4, WT1, GATA4, GATA1, SCF, FGF2* and *AR* were expressed in the isolated cells (Fig. 1A), whereas *VASA* was undetected in these cells (Fig. 1A). PCR with water but without cDNA served as a negative control, and PCR with *ACTB* was used as loading controls for total RNA (Fig. 1A). The purity of isolated human cells was determined using immunocytochemistry. As shown in Fig. 1B–I, more than 96% of the cells were positive for WT1 (Fig. 1B), BMP4 (Fig. 1C), SOX9 (Fig. 1D), GDNF (Fig. 1E), SCF (Fig. 1F), OCLN (Fig. 1G), ZO1 (Fig. 1H), and VIM (Fig. 1I). Replacement of primary antibodies with PBS served as a negative control, and no immunostaining was observed in these cells (Fig. 1J). Considered together, these results suggest that the isolated cells were human Sertoli cells phenotypically.

### Expression of BMP6 and Its Receptors in Adult Human Sertoli Cells

**Figure 2:**
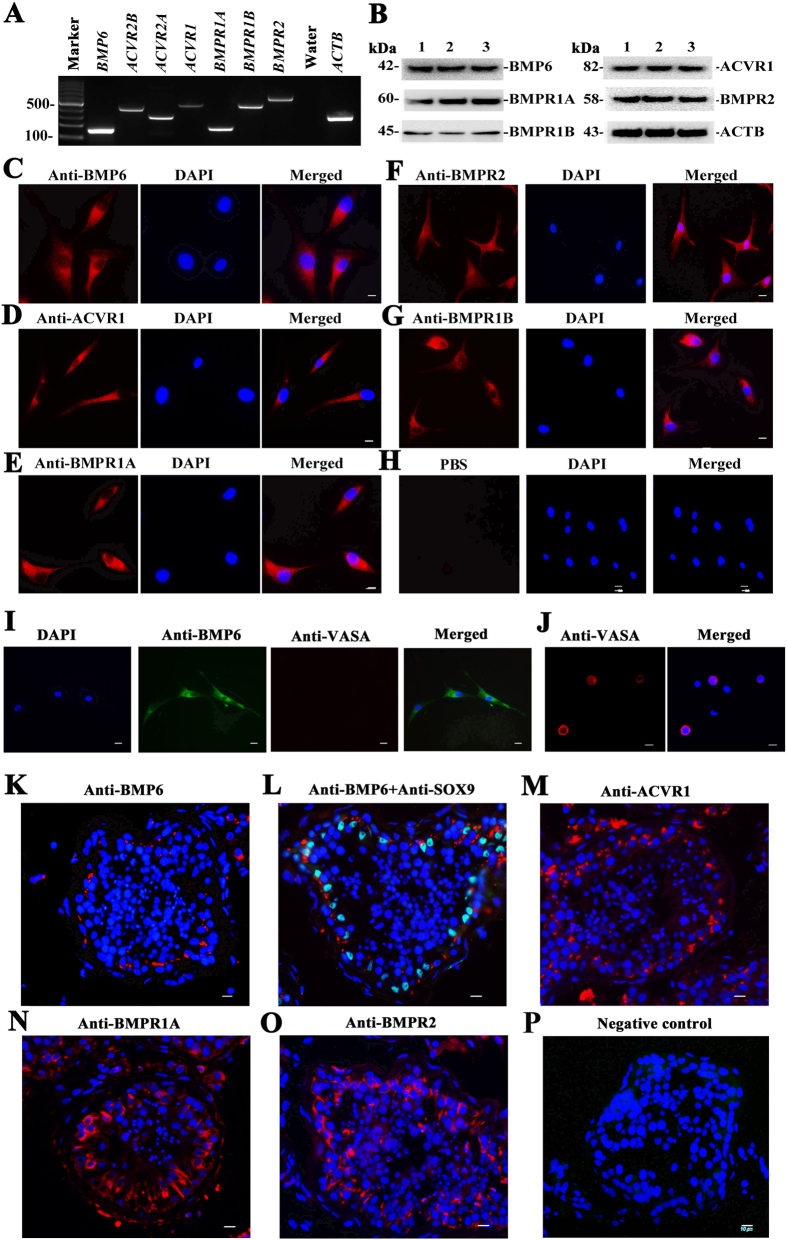
The expression of BMP6 and its receptors, including ACVR1, BMPR1A, BMPR1B, ACVR2A, ACVR2B and BMPR2 in human Sertoli cells and testis. (**A**) RT-PCR displayed the expression of *BMP6* and its receptors in human Sertoli cells. Water without DNA served as a negative control and *ACTB* was employed as a loading control for total RNA. (**B**) Western blots showed the expression of BMP6, ACVR1, BMPR1A, BMPR1B and BMPR2 in the isolated human Sertoli cells of three independent samples. Notes: 1, 2, 3 indicated three different samples. (**C–H**) Immunocytochemistry illustrated the expression of BMP6 (**C**), ACVR1 (**D**), BMPR1A (**E**), BMPR2 (**F**), and BMPR1B (**G**) in the isolated human Sertoli cells. Replacement of primary antibodies with PBS served as a negative control (**H**). (**I**) Double immunostaining showed the expression of BMP6 and VASA in human Sertoli cells. (**J**) Immunofluorescence showed the presence of VASA in male germ cells of OA patients. (**K–P**) Immunofluorescence revealed cellular localization of BMP6 (**K**), BMP6 and SOX9 (**L**), ACVR1 (**M**), BMPR1A (**N**) and BMPR2 (**O**) in human testis. Replacement of primary antibodies with PBS served as a negative control (**P**). Scale bars in C-P = 10 μm.

After isolation and identification of adult human Sertoli cells, total RNA was extracted from these cells of OA patients. BMP6 ligand and its multiple receptors, including ACVR1, BMPR1A, BMPR1B, ACVR2A, ACVR2B, and BMPR2^21^, were determined in human Sertoli cells. RT-PCR showed that transcripts of *BMP6* and its receptors *ACVR1, BMPR1A, BMPR1B, ACVR2A, ACVR2B* and *BMPR2* were expressed in the freshly isolated human Sertoli cells (Fig. 2A). PCR with water but without cDNA served as a negative control, and PCR with *ACTB* was used as loading controls for total RNA (Fig. 2A). Western blots further showed that BMP6 and its receptors, including ACVR1, BMPR1A, BMPR1B and BMPR2, were present in the isolated human Sertoli cells (Fig. 2B). Furthermore, immunocytochemical staining displayed that BMP6 (Fig. 2C) and its multiple receptors, including ACVR1 (Fig. 2D), BMPR1A (Fig. 2E), BMPR2 (Fig. 2F), and BMPR1B (Fig. 2G), were present in the isolated human Sertoli cells. Replacement of primary antibodies with PBS served as a negative control, and no immunostaining was observed in these cells (Fig. 2H). Double immunostaining showed that BMP6 was expressed in the isolated cells whereas VASA was undetected in these cells (Fig. 2I). As a positive control, VASA immunostaining was observed in some human male germ cells from the testicular tissues after two-step enzymatic digestion (Fig. 2J). Immunofluorescence revealed that BMP6 was present in human Sertoli cells rather than male germ cells in human testis (Fig. 2K), and BMP6 was coexpressed with SOX9 (a marker for Sertoli cells^22^) in human Sertoli cells (Fig. 2L). ACVR1 (Fig. 2M), BMPR1A (Fig. 2N) and BMPR2 (Fig. 2O) were expressed in human Sertoli cells as well as human spermatogonia and spermatocytes. Replacement of primary antibodies with PBS served as a negative control, and no immunostaining was observed in human testis (Fig. 2P).

### BMP6 Promoted the Proliferation and DNA Synthesis But Inhibited the Apoptosis of Human Sertoli Cells

**Figure 3:**
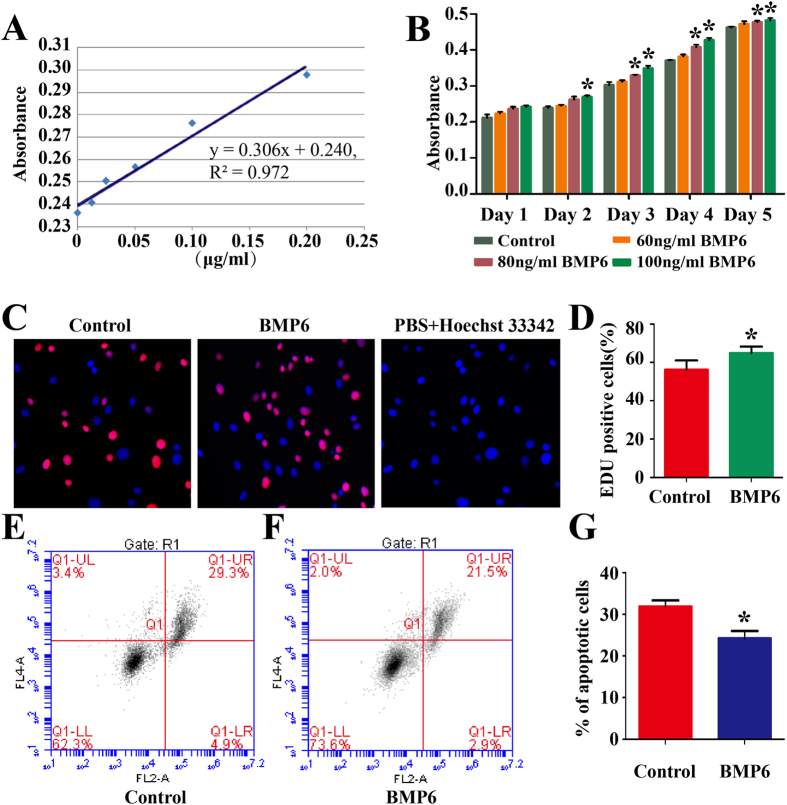
The effect of BMP6 on the proliferation, DNA synthesis and apoptosis of adult human Sertoli cells. (**A,B**) CCK-8 assay showed the standard curve (**A**) and growth curve of human Sertoli cells treated with various doses of human recombinant BMP6 factor (**B**) for 5 days. (**C,D**) Immunocytochemical staining showed the EDU incorporation in human Sertoli cells without treatment (control) or treated with 100 ng/ml BMP6 for 48 hours. Cell nuclei were counterstained with Hoechst 33342. The percentages of EDU-positive cells were counted out of 500 total cells from three independent experiments. (**E–G**) Annexin V-APC/PI and flow cytometry analysis showed the apoptosis in human Sertoli cells by 100 ng/ml BMP6 or 0.1% BSA (control) at 72 hours. *Indicated statistically significant differences (p < 0.05) between the control and BMP6-treated group.

We probed the effect of BMP6 on the proliferation and apoptosis of human Sertoli cells. CCK-8 assay was performed in human Sertoli cells using recombinant human BMP6 factor and RNA interference^8^. Standard curve indicated a good correlation between the absorbance and the concentrations of BMP6 in human Sertoli cells (Fig. 3A). CCK-8 assay showed that recombinant human BMP6 promoted the proliferation of human Sertoli cells in dose- and time- dependent ways (Fig. 3B). BMP6 induced a significant increase of cell number of these cells at 100 ng/ml for 5 days. We further examined the influence of BMP6 on DNA synthesis in human Sertoli cells utilizing the EDU incorporation assay. Compared to the control (56.2% ± 4.8% of EDU-positive cells), BMP6 increased the EDU-positive cells up to 64.8% ± 3.5% (Fig. 3C,D), reflecting that BMP6 promotes DNA synthesis of human Sertoli cells. We also probed the influence of BMP6 on the apoptosis of human Sertoli cells, and we found that the average percentage of apoptosis of Sertoli cells was decreased by 7.7% ± 1.9% after treatment with BMP6 at 72 hours (Fig. 3F,G), compared with control group (Fig. 3E,G).

**Figure 4:**
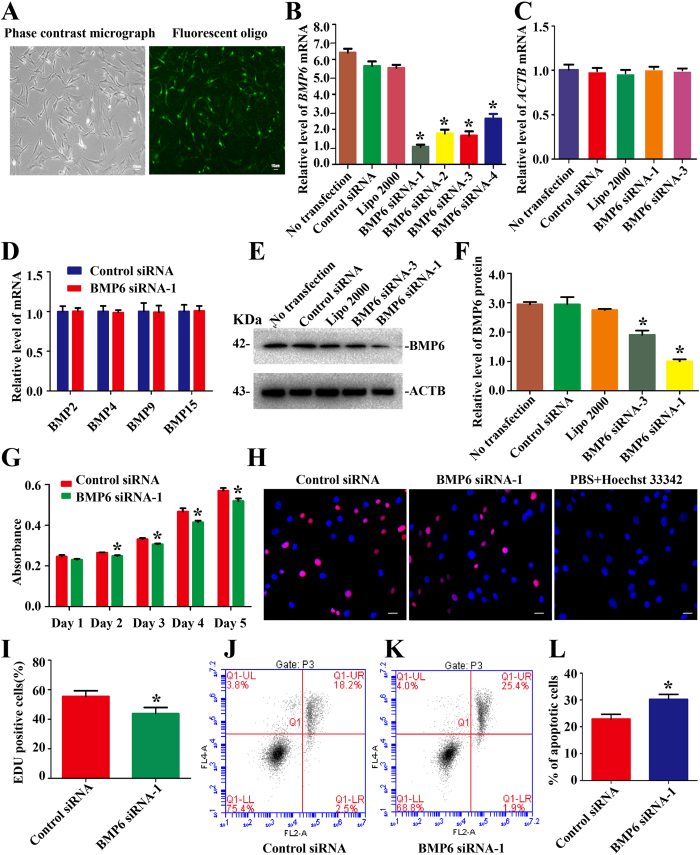
Transfection of BMP6 siRNAs and the influence of BMP6 knockdown on the proliferation and apoptosis of human Sertoli cells. (**A**) The transfection with FAM-labeled siRNA at 6 hours showed the transfection efficiency of BMP6 siRNAs. Scale bars in A = 10 μm. (**B**) Real-time PCR showed the transcription of *BMP6* in human Sertoli cells at 24 hours after transfection with BMP6 siRNAs and control siRNA. (**C**) Real-time PCR displayed *ACTB* transcript in human Sertoli cells at 24 hours after transfection with BMP6 siRNA-1 and -3, control siRNA, lipofectamine 2000, or no transfection. (**D**) Real-time PCR revealed the expression of *BMP2, BMP4, BMP9*, and *BMP15* in human Sertoli cells treated with BMP6 siRNA-1 and the control siRNA. (**E,F**) Western blots demonstrated the BMP6 protein expression in human Sertoli cells at 48 hours after transfection with control siRNA or BMP6 siRNA-1 or BMP6 siRNA-3. ACTB served as a loading control of proteins. (**G**) CCK-8 assay showed the growth curve of human Sertoli cells after transfection of control siRNA or BMP6 siRNA-1 for 1 to 5 days. (**H,I**) EDU incorporation assay showed the EDU-positive cells in human Sertoli cells after transfection of control siRNA or BMP6 siRNA-1. Cell nuclei were counterstained with Hoechst 33342. The percentages of EDU-positive cells were counted out of 500 total cells from three independent experiments. Scale bars in H = 10 μm. (**J–L**) Annexin V-APC/PI and flow cytometry analysis revealed apoptosis in human Sertoli cells at 48 hours after transfection of control siRNA (**J**) or BMP6 siRNA-1 (**K**). *Denoted statistically significant differences (p < 0.05) between the control and BMP6 siRNA-1-treated group.

In addition, we utilized small RNAs to elucidate the effect of endogenous BMP6 on the growth of human Sertoli cells. To improve the efficiency of BMP6 knockdown, we designed four pairs of BMP6 siRNAs targeting different regions of BMP6 mRNA. FAM-labeled siRNA represented the transfection efficiency. At 6 hours after transfection, the transfection efficiency of siRNA in human Sertoli cells was over 80% (Fig. 4A). The viability of human Sertoli cells after transfecting BMP6 siRNAs was about 98%, as assessed by trypan blue exclusion assay (data not shown). Quantitative real-time PCR revealed that BMP6 siRNA-1, -2, -3, and-4 apparently decreased *BMP6* mRNA in Sertoli cells at 24 hours after transfection (Fig. 4B), while the interfering effect of BMP6 siRNA-1 was the most prominent (The knockdown rates of BMP6 siRNAs were shown in Table S3). Real-time PCR showed that no obvious difference existed in the transcription of *ACTB* of human Sertoli cells after transfection with BMP6 siRNAs, control siRNA, lipofectamine 2000, or no transfection (Fig. 4C), thus confirming that *ACTB* could be used as a good control. Notably, there was no significant change of *BMP2, BMP4, BMP9*, and *BMP15* transcripts by BMP6 siRNA-1 and the control siRNA (Fig. 4D), thus verifying the specific silencing of BMP6 siRNA-1 in human Sertoli cells. Western blots revealed that BMP6 siRNA-1 reduced the expression of BMP6 protein by 300.0% ± 27.7% at 48 hours after transfection (Fig. 4E,F). Therefore, we chose BMP6 siRNA-1 to investigate the effect of BMP6 knockdown on the proliferation in human Sertoli cells. CCK-8 assay was executed from 24 hours to 120 hours after transfection of BMP6 siRNA-1 in human Sertoli cells, and BMP6 siRNA-1 evidently reduced the proliferation in human Sertoli cells at 48 hours to 120 hours (Fig. 4G). Similarly, the percentage of EDU-positive cells was reduced in human Sertoli cells with BMP6 siRNA-1 treatment compared with the control siRNA (55.4% ± 3.9% vs. 43.8% ± 4.2%, n = 5) at 48 hours after transfection (Fig. 4H,I). Additionally, we found that the apoptosis of Sertoli cells was increased by 10.7% ± 2.6% after BMP6 siRNA-1 transfection at 48 hours (Fig. 4J–L). Collectively, these data indicate that BMP6 stimulates DNA synthesis and the proliferation and inhibits the apoptosis of human Sertoli cells.

### BMP6 Knockdown Diminished the Function of Human Sertoli Cells

**Figure 5:**
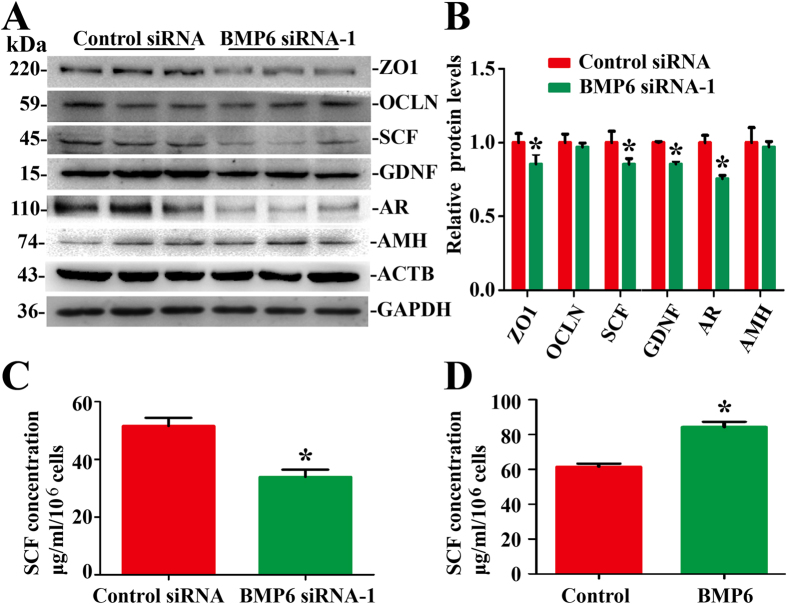
The influence of BMP6 on the function of human Sertoli cells. (**A**) Western blots demonstrated ZO1, OCLN, SCF, GDNF, AR and AMH proteins in human Sertoli cells at 48 hours after transfection with control siRNA or with BMP6 siRNA-1. ACTB and GAPDH served as loading controls of proteins. Full length blots of ZO1, OCLN and GDNF were presented in Supplementary Figure B. (**B**) The relative expression of ZO1, OCLN, SCF, GDNF, AR and AMH in human Sertoli cells at 48 hours after transfection with BMP6 siRNA-1 or control siRNA after normalization to the signals of their respect loading control. *Indicated statistically significant differences (p < 0.05) between control and BMP6 siRNA-treated group. (**C,D**) ELISA showed SCF secretion by BMP6 siRNA-1 (**C**) and BMP6 (**D**) in human Sertoli cells. *Indicated statistically significant differences (p < 0.05) between the control siRNA and BMP6 siRNA-1, or the control and BMP6-treated group.

We next utilized small RNAs to investigate the effect of endogenous BMP6 on the function of human Sertoli cells. ZO1 and OCLN are the indispensable components of tight junction^23^. Western blots revealed that the level of ZO1 protein was reduced by 22.9% ± 3.2% at 48 hours after transfection of BMP6 siRNA-1 (Fig. 5A,B), while OCLN and AMH levels were not significantly changed (Fig. 5A,B). Sertoli cells can secrete some important growth factors, including GDNF and SCF, which regulate the self-renewal and differentiation of spermatogonial stem cells. Western blots showed that the production of SCF, GDNF and AR (androgen receptor) was reduced in human Sertoli cells at 48 hours after transfection of BMP6 siRNA-1 (Fig. 5A,B). We further measured SCF secretion in human Sertoli cells using ELISA, and the expression of SCF was significantly reduced by 17.7 ± 3.3 μg/ml/10^6^cells by BMP6 siRNA-1 compared to the control siRNA at 48 hours after transfection (Fig. 5C). In contrast, the secretion of SCF was increased to 81.6 ± 9.0 μg/ml/10^6^cells by BMP6 (Fig. 5D) at 72 hours, compared with control group (61.5 ± 3.0 μg/ml/10^6^cells). Taken together, these data implicate that BMP6 stimulates the function of human Sertoli cells.

### BMP6 Activated Smad2/3 and Cyclin D1 Signaling Pathway in Human Sertoli Cells

**Figure 6:**
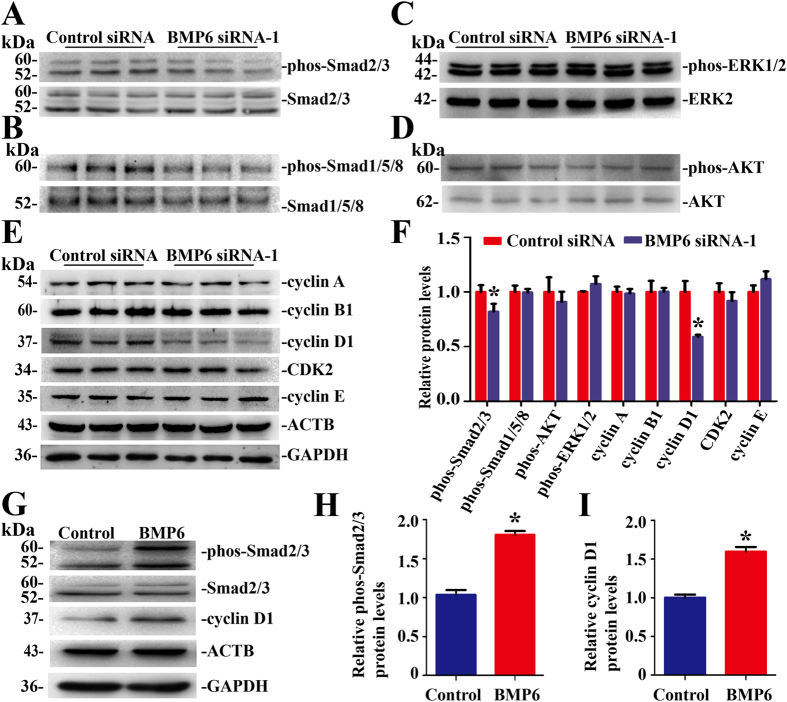
BMP6 activated Smad2/3 and cyclin D1 pathway in human Sertoli cells. (**A**) Western blots showed the change of phos-Smad2/3 in human Sertoli cells at 48 hours after transfection with control siRNA or with BMP6 siRNA-1. Smad2/3 was used as a loading control of total proteins. Full length blots of phos-Smad2/3 and ACTB were presented in Supplementary Figure C. (**B–D**) Western blots revealed the expression of phosphorylation of Smad1/5/8 (**B**), phosphorylation of ERK1/2 (**C**), and phosphorylation of AKT (**D**) in human Sertoli cells at 48 hours after transfection with control siRNA or with BMP6 siRNA-1. Smad1/5/8, ERK2, and AKT served as loading controls for total proteins, respectively. Full length blots of phos-ERK1/2 and ERK were presented in Supplementary Figure D. (**E**) Western blots showed the expression of cyclin A, cyclin B1, cyclin D1, CDK2 and cyclin E in human Sertoli cells at 48 hours after transfection with control siRNA or with BMP6 siRNA-1. ACTB and GAPDH served as a loading control of total proteins. Full length blots of cyclin D1, CDK2, cyclin E and ACTB were presented in Supplementary Figure E. (**F**) The relative expression of phos-Smad2/3, phos-Smad1/5/8, phos-AKT, phos-ERK1/2, cyclin A, cyclin B1, cyclin D1, CDK2, and cyclin E in human Sertoli cells at 48 hours after transfection with BMP6 siRNA-1 or control siRNA after normalization to the signals of their respect loading control. *indicated statistically significant differences (p < 0.05) between the control siRNA and BMP6 siRNA-1-treated group. (**G–I**) Western blots demonstrated the levels of phos-Smad2/3 and cyclin D1 by BMP6 and BSA (control) in human Sertoli cells.

We further explored the signaling pathways and transcription factors activated by BMP6 in human Sertoli cells. To this end, we detected the expression of phos-Smad2/3, phos-Smad1/5/8, phos-AKT and phos-ERK1/2. Western blots revealed that phos-Smad2/3 was reduced by 25.0% ± 2.5% in human Sertoli cells after transfection of BMP6 siRNA-1 compared with control siRNA (Fig. 6A and F). No significant changes were observed in phos-Smad1/5/8, phos-AKT, or phos-ERK1/2 in human Sertoli cells after transfection of BMP6 siRNA-1 (Fig. 6B–D and F). Furthermore, we checked the expression of various cell cycle progression proteins, including cyclin A, B1, D1, E and CDK2, to address which cell cycle proteins were affected by endogenous BMP6. Western blots demonstrated that the level of cyclin D1 was reduced by 41.1% ± 3.2% in human Sertoli cells transfected with BMP6 siRNA-1 compared with control siRNA at 48 hours (Fig. 6E,F). In contrast, no obvious changes were seen in the levels of cyclin A, B1, E, and CDK2 by BMP6 siRNA-1 compared to control siRNA (Fig. 6E,F). Additionally, Western blots revealed that BMP6 increased the level of phos-Smad2/3 by 70.9% ± 4.9% at 30 min and cyclin D1 at 24 hours by 56.3% ± 3.2% in human Sertoli cells (Fig. 6G–I). Therefore, BMP6 activates Smad2/3 pathway and enhances the level of cyclin D1 in human Sertoli cells.

### BMP6 Activated Transcription Factors DACH1 and TFAP2A in Human Sertoli Cells

**Figure 7:**
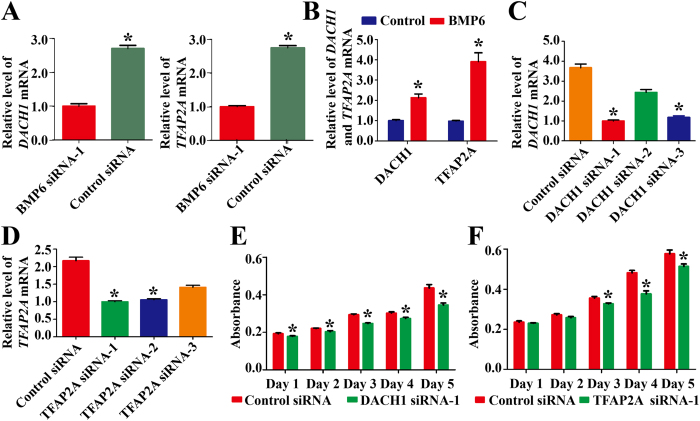
The effect of transcription factor DACH1 and TFAP2A on the regulation of the growth in human Sertoli cells. (**A**) Real-time PCR showed the expression of *DACH1* and *TFAP2A* in human Sertoli cells at 48 hours after transfection with BMP6 siRNA-1 and control siRNA. (**B**) Real-time PCR displayed the expression of *DACH1* and *TFAP2A* in human Sertoli cells without or with treatment of BMP6. (**C,D**) Real-time PCR demonstrated the *DACH1* and *TFAP2A* expression in human Sertoli cells at 24 hours after transfection with control siRNA or DACH1 siRNAs or TFAP2A siRNAs. (**E-F**) CCK-8 assay displayed the growth curve of human Sertoli cells after transfection of control siRNA or DACH1 siRNA-1 or TFAP2A siRNA-1 for 1 to 5 days. *indicated statistically significant differences (p < 0.05) between control siRNA and siRNA-treated group.

To identify the targets of BMP6 in regulating proliferation of human Sertoli cells, the changes of transcription profile were measured in human Sertoli cells transfected with BMP6 siRNA-1 at 48 hours. It had been reported that DACH1, a dachshund family transcription factor, inhibits Smad2/3 signaling^24^, while TFAP2A (transcription factor AP2 alpha) strengthens the binding of Smad2/3 to target promoters and affects transcriptional response^25^. Real-time PCR revealed that the transcription of *DACH1* and *TFAP2A* was decreased by 2.7 ± 0.2 fold and 2.8 ± 0.1 fold, respectively, in human Sertoli cells treated with BMP6 siRNA-1 compared with control siRNA (Fig. 7A). Furthermore, we found that BMP6 enhanced the levels of *DACH1* by 2.1 ± 0.1 fold and *TFAP2A* by 3.9 ± 0.2 fold in human Sertoli cells (Fig. 7B).

Finally, we probed the function of BMP6 targets in human Sertoli cells. We silenced the expression of DACH1 and TFAP2A to detect the effect of endogenous DACH1 and TFAP2A on the growth of human Sertoli cells. Similarly, we designed three pairs of siRNAs against these genes to improve the efficiency of the knockdown of DACH1 and TFAP2A siRNAs. The viability of human Sertoli cells after transfecting DACH1 and TFAP2A siRNAs was around 97%, as evaluated by trypan blue exclusion assay (data not shown). Quantitative real-time PCR revealed that DACH1 siRNA-1 reduced the *DACH1* mRNA by 3.7 ± 0.2 fold in Sertoli cells at 48 hours after transfection (Fig. 7C), and TFAP2A siRNA-1 significantly diminished the *TFAP2A* mRNA by 2.2 ± 0.2 fold in Sertoli cells at 48 hours after transfection (Fig. 7D) (The knockdown rates of DACH1 siRNAs and TFAP2A siRNAs were shown in Tables S4 and S5). CCK-8 assay was executed in human Sertoli cells after transfection of DACH1 siRNA-1 and TFAP2A siRNA-1. The proliferation of human Sertoli cells was obviously decreased by DACH1 siRNA-1 and TFAP2A siRNA-1 at 24 hours and 72 hours to 120 hours, respectively (Fig. 7E,F). At 120 hours after transfection of DACH1 siRNA-1 and TFAP2A siRNA-1, the absorbance of human Sertoli cells was decreased from 0.44 ± 0.04 to 0.36 ± 0.02 and from 0.58 ± 0.04 to 0.51 ± 0.02, respectively (Fig. 7E,F). Together, these data suggest that the proliferation of human Sertoli cells is in part regulated by DACH1 and TFAP2A transcription factors.

## Discussion

Sertoli cells play critical roles in regulating normal spermatogenesis, since they are the main component of blood-testis barrier, which provides a suitable environment for male germ cell development. In addition, Sertoli cells offer nutritional and immunological support for male germ cells^26^. Abnormal number and/or quality of Sertoli cells can lead to dyszoospermia^27^. BMP6 has been suggested to be involved in controlling the process of embryogenesis, hematopoiesis, skeletal formation and neurogenesis. Here we have demonstrated that BMP6 promotes the proliferation and DNA synthesis of human Sertoli cells and inhibits their apoptosis.

We first examined the expression and cellular localization of BMP6 as well as its multiple receptors in human testis. We found that BMP6 ligand and its multiple receptors, namely ACVR1, BMPR1A, BMPR1B, ACVR2A, ACVR2B and BMPR2, were expressed in human Sertoli cells. We further showed that BMP6 was present in human Sertoli cells but not in male germ cells, whereas its receptors were detected in human Sertoli cells and some types of human male germ cells (e.g. spermatogonia and spermatocytes), reflecting that Sertoli cells are the main source of BMP6 within human seminiferous tubules. These results suggest that BMP6 acts via an autocrine pathway in human Sertoli cells, whereas it regulates the fate determinations of human male germ cells through a paracrine manner.

We next explored the function of BMP6 in regulating human Sertoli cells. Adult Sertoli cells have previously been considered the terminally differentiated cells that are unable to divide or proliferate. Nevertheless, this concept has recently been challenged^28^, because Sertoli cells from adult hamster^29^ and human^30^ can regain proliferative ability *in vitro*. We found that exogenous BMP6 promoted the proliferation of human Sertoli cells. Furthermore, we used RNA interference to probe the role of endogenous BMP6 in human Sertoli cells. CCK-8 assay and EDU incorporation assays demonstrated that the growth of human Sertoli cells was obviously reduced when they were treated with BMP6 siRNA-1 compared with the control siRNA. Cell division was decreased in human Sertoli cells after knockdown of endogenous BMP6. Considered together, these data implicate that BMP6 stimulates the division and proliferation of human Sertoli cells. In addition, we compared the apoptotic percentages in human Sertoli cells when treated with exogenous BMP6 or knockdown of the endogenous BMP6, and we found that BMP6 could inhibit the apoptosis of human Sertoli cells. These data suggest that BMP6 suppresses the apoptosis of human Sertoli cells.

ZO1 is a main component of tight junction complexes that provide a stable microenvironment for germ cells and act as an immunologic barrier to protect male germ cells with autoimmunity^23^. Notably, we observed that ZO1 protein was reduced in human Sertoli cells by BMP6 knockdown. In addition to the change of structural protein ZO1, the production of GDNF and SCF was significantly reduced by BMP6 siRNA in human Sertoli cells. It has been reported that GDNF can promote the self-renewal of spermatogonial stem cells *in vitro* and *in vivo*^31^,^32^,^33^,^34^. Previous studies have demonstrated that SCF is required for the proliferation and differentiation of spermatogonia by binding its receptor KIT via activating the PI3K/Akt pathway^35^,^36^. We found that BMP6 regulated the secretion of GDNF and SCF in human Sertoli cells. Androgen receptor (AR), another factor expressed in Sertoli cells, is crucial for the development and the maintenance of male germ cells in testis^37^. Sertoli cells specific AR-knockout mice have smaller testes with hypotestosteronemia, arrested spermatogenesis, no mature sperm in epididymides, and defective seminiferous tubule development^38^,^39^,^40^,^41^,^42^,^43^. In this study, a significant reduction of AR was seen in human Sertoli cells when BMP6 was knocked down. Together, these data mentioned above reflect that BMP6 plays an important role in maintaining the function of human Sertoli cells.

We further asked which signaling pathways and transcription factors were activated by BMP6 in human Sertoli cells. BMP6 signaling pathway starts by binding its receptors and followed by the phosphorylation of Smads. The Smads combining with Smad4 (co-Smad) enters the nucleus and coordinates with other transcription factors to affect target gene expression^44^. Notably, we found that the level of phos-Smad2/3 was significantly enhanced by BMP6 and decreased by BMP6 siRNA in human Sertoli cells, whereas no obvious changes was observed in phos-Smad1/5/8, phos-ERK1/2 or phos-AKT in human Sertoli cells by the knockdown of BMP6. These results suggest that BMP6 activates only Smad2/3 pathway but not Smad1/5/8, ERK1/2 or AKT pathways in human Sertoli cells. We observed that the expression of cyclin D1 protein was increased by BMP6 and reduced by BMP6 siRNAs, whereas no significant changes in the expression levels of cyclin A, B1, E and CDK2, indicating that the level of cyclin D1 is increased by BMP6 in human Sertoli cells. Furthermore, we detected the transcription factors of BMP6 by real-time PCR after the knockdown of BMP6. We found that the levels of DACH1 and TFAP2A were reduced by BMP6 siRNA and increased by BMP6. DACH1 proteins contain a domain conserved with the proto-oncogenes Ski and Sno, and DACH1 suppresses gastric cancer cell proliferation by inhibiting TGF-β signal pathway activity through binding Smad4^45^. In this study, we found that the proliferation of human Sertoli cells was reduced when the DACH1 was silenced by RNAi. These data imply that transcription factor DACH1 is the target of BMP6 in regulating the growth of human Sertoli cells. TFAP2A is a DNA-binding transcription factor for terminal differentiation^46^,^47^, and it is both positive and negative factor in the regulation of TGF-β signaling pathway^48^. TFAP2A is significantly enriched in Smad2/3 promoter regions, and it assists Smad2 and Smad3 to transmit the transforming of TGF-β/Smad signal from the plasma membrane to the nucleus^25^. In the current study, we found that the proliferation of human Sertoli cells was reduced after silencing TFAP2A, which was consistent with the function of BMP6. Therefore, TFAP2A is the targeting transcription factor of BMP6 in regulating the growth of human Sertoli cells.

In summary, we have identified BMP6 as an autocrine factor in mediating the fate decisions of adult human Sertoli cells. Specifically, BMP6 promotes DNA synthesis and proliferation and suppresses the apoptosis of adult human Sertoli cells; BMP6 enhances the expression of SCF, GDNF, AR, and tight junction protein ZO1. Notably, we have demonstrated that BMP6 activates the Smad2/3/cyclin D1 pathway and DACH1 and TFAP2A transcription factors. This study thus provides new insights into molecular mechanisms underlying human Sertoli cell development.

## Methods

### Procurement of Human Testicular Tissues from OA Patients

Testicular tissues were obtained from 32 OA patients who underwent microdissection and sperm extraction at Ren Ji Hospital, School of Medicine, Shanghai Jiao Tong University. All OA patients were examined histologically and they had normal spermatogenesis. The obstruction of these patients was caused by inflammation and/or vasoligation.

### Ethics Statement

The methods of this study were approved by the Institutional Ethical Review Committee of Ren Ji Hospital, School of Medicine, Shanghai Jiao Tong University (license number of ethics statement: 2012–01). All experimental protocols were performed in accordance with relevant guidelines and regulations of the Institutional Ethical Review Committee of Ren Ji Hospital. An informed consent of testis tissues used for research only was obtained from each OA patient.

### Isolation of Human Sertoli cells from OA Patients

Two-step enzymatic digestion was utilized to isolate male germ cells from human testis tissues. Testicular tissues from OA patients were washed three times in Dulbecco modified Eagle medium (DMEM) (Gibco, Grand Island, USA) with 2% antibiotics containing penicillin and streptomycin (Gibco). The testis tissues were cut into pieces and incubated with 10 mL DMEM containing 2 mg/mL type IV Collagenase (Gibco) and 10 μg/mL DNase I (Sigma, Saint Louis, USA) in oscillating water bath at 34 °C at 100 rpm for 10–15 min. The seminiferous tubules were washed extensively with DMEM to remove Leydig cells and myoid cells and they were collected via the sedimentation. Human seminiferous tubules were incubated with 10 mL DMEM including 2.5 mg/mL type IV collagenase, 2 mg/ml Hyaluronidase (Sigma), 2 mg/ml Trypsin (Sigma) and 10 μg/ml DNase I in oscillating water bath at 34 °C, 100 rpm for 10–15 min. The suspension was centrifuged at 300 g for 5 min and the supernatant was removed. The precipitant was resuspended in DMEM/F-12 with 10% FBS and followed by being filtered using a 40 μm cell strainer and planted in a 10 cm dish pre-coated with gelatin. The cells were cultured in DMEM/F-12 (Gibco) supplemented with 10% FBS at 34 °C in 5% CO_2_ for 3 hours. After incubation, the medium containing male germ cells was removed, and Sertoli cells were attached to culture dishes.

### Trypan Blue Staining

To assess the viability of freshly isolated human Sertoli cells and the cells after transfecting BMP6 siRNAs, DACH1 siRNAs, or TFAP2A siRNAs, trypan blue staining was performed. Briefly, the cells were incubated with 0.4% trypan blue, and viability of these cells was evaluated by percentage of the cells excluding trypan blue staining and total cells counted.

### Culture of Human Sertoli Cells from OA Patients

Freshly isolated human Sertoli cells were seeded at a density of 2 × 10^5^ cells/cm^2^ in 100 mm × 20 mm dish and cultured in DMEM/F-12 supplemented with 10% FBS and 1% antibiotics containing penicillin and streptomycin (Gibco) for overnight. To exclude the effect of FBS on the role of BMP6 in controlling the proliferation of human Sertoli cells, the medium was changed to DMEM/F-12 supplemented with 1% FBS which didn’t cause apoptosis of these cells. The cells were treated without or with 60, 80 or 100 ng/ml of BMP6 (Peprotech, Rocky Hill, USA), or BMP6 siRNAs(Genepharma, Suzhou, Jiangsu, China), DACH1 siRNAs(Genepharma), TFAP2A siRNAs (Genepharma) or control siRNA (Genepharma) pursuant to the RNA interference protocol as described below.

### RNA Extraction and Reverse Transcription Polymerase Chain Reaction (RT-PCR) and Quantitative Real-Time PCR

Total RNA was extracted from freshly isolated human Sertoli cells using Trizol (Takara, Kusatsu, Japan), and the quality and concentrations of total RNA from human Sertoli cells were measured by Nanodrop (Thermo). The ratios of A_260_/A_280_ of total RNA were set as 1.9–2.0 to ensure good purity. In addition, RNA integrity and quality were measured using an Agilent 2100 Bioanalyzer. RNA samples with RNA Integrity Number (RIN) values of more than 7.0 were used for RT-PCR and real-time PCR. Reverse transcription (RT) of total RNA was conducted using the First Strand cDNA Synthesis Kit (Thermo Scientific, USA), and PCR of the cDNA was carried out according to the protocol as described previously^49^. We detected a number of genes, including *WT1* (Wilms tumor1), *SOX9* (Sex Determining Region Y-Box 9), *BMP4, GDNF, GATA1* (GATA binding protein 1), *GATA4, SCF, FGF2, AR, VASA, BMP6, ACVR1, BMPR1A, BMPR1B, ACVR2A, ACVR2B, BMPR2* and *ACTB*. The primer sequences of these genes were designed and listed in Table S1. The PCR reactions started at 94 °C for 2 min and were performed in terms of the following conditions: denaturation at 94 °C for 30 sec, annealing at 55–60 °C for 45 sec as listed in Table S1, and elongation at 72 °C for 45 sec, for 35 cycles. The samples were incubated for an additional 5 min at 72 °C. PCR with water but without cDNA served as a negative control. PCR products were separated by electrophoresis with 2% agarose gel and they were visualized with ethidium bromide. The band intensities of PCR products were analyzed using chemiluminescence (Chemi-Doc XRS, Bio-Rad).

Quantitative real-time PCR reactions were performed using Power SYBR^®^ Green PCR Master Mix (Applied Biosystems, Woolston Warrington, UK) and a 7500 Fast Real-Time PCR System (Applied Biosystems, Carlsbad, CA, USA). To quantify the PCR products, the comparative Ct (threshold cycle) method was used as described previously^50^. The threshold of cycle values of genes was normalized against the threshold value of human housekeeping gene *ACTB* [ΔC_T_ = C_T_( *gene*)− C_T(*ACTB*)_], and the relative expression of genes in treated group to the control was calculated by formula 2^−ΔΔCT^ [ΔΔC_T_ = ΔC_T(treated)_−ΔC_T (control)_]. The primer pairs of detected genes were listed in Table S1. The Ct values and a series of five-fold dilutions of template cDNA of human Sertoli cells were utilized for drawing the standard curves, and the slope of each standard curve was used to calculate the efficiency (E) of gene primers using the formulae: E = 10^(−1/slope)^-1, according to the method described previously^51^,^52^,^53^,^54^.

### Immunocytochemistry

For immunocytochemistry, freshly isolated and cultured human Sertoli cells were fixed with 4% paraformaldehyde (PFA) for 30 min, and they were washed three times with cold phosphate-buffered saline (PBS) (Medicago, Uppsala, Sweden) and permeabilized with 0.3% triton-X 100 (Sigma) for 5 min. After extensive washes with PBS, the cells were blocked in 5% bovine serum albumin (BSA) (Sigma) for 1 hour. These cells were incubated with primary antibodies, including WT1, BMP4, SOX9, GDNF, SCF, OCLN, ZO1, VIM, BMP6, VASA, ACVR1, BMPR1A, BMPR1B, and BMPR2, overnight at 4 °C. The detailed information of primary antibodies was listed in Table S2, and the specificity of these antibodies was evaluated previously^8^ and proven to be perfect. After extensive washes with PBS, the cells were incubated with the secondary antibody, namely IgGs conjugated with fluorescein isothiocyanate (FITC) (Sigma) or rhodamine-conjugated IgG (Sigma), at a 1:200 dilution for 1 hour at room temperature. To label the nuclei of the cells, DAPI (4, 6-diamidino-2-phenylindole) was employed, and the images were captured with a Nikon microscope.

### Immunofluorescence

Testis sections of OA patients were deparaffinized by xylene three times, and hydrated with a series of graded alcohol and treated with 3% H_2_O_2_ (Boster Biological Technology, Guangzhou, China) to block the endogenous peroxidase activity. After blocking with 5% BSA for 1 hour, the sections were incubated with primary antibodies, including BMP6, SOX9, ACVR1, BMPR1A and BMPR2 (Table S2). After extensive washes with PBS for 30 min, Fluorescein isothiocyanate (FITC)- or rhodamine-conjugated IgG was used as secondary antibody, and DAPI was used to label the nuclei. Replacement of primary antibodies with PBS was used as negative controls and images were observed under a Nikon microscope.

### Western Blots

Human Sertoli cells without or with BMP6, control siRNAs, BMP6 siRNA, DACH1 siRNA, and TFAP2A siRNA treatment were lysed with RIPA buffer (BiotechWell, Guangzhou, Shanghai, China) for 30 min on ice. Cell lysates were cleared by centrifugation at 12,000 g for 15 min at 4 °C, and the protein concentrations were measured by BCA kit (DingGuo ChangShengBiotech, Beijing, China). Thirty micrograms of cell lysate from each sample were used for 4–12% SDS-PAGE (Bio-Rad Laboratories), and Western blots were performed according to the protocol as described previously^33^. In brief, samples were resolved in the XCell SureLock Novex Mini-Cell apparatus (Invitrogen, Carlsbad, USA) and transferred to nitrocellulose membranes for 2 h at room temperature. The membranes were washed with TBS containing 0.1% Tween (TBS-T) (DingGuo ChangShengBiotech) and were blocked with 5% nonfat dry milk in TBS-T for 1 h at room temperature. After extensive washes with TBS-T, the membranes were incubated with the chosen antibodies. The primary antibodies included BMP6, ACVR1, BMPR1A, BMPR1B, BMPR2, ZO1, OCLN, SCF, GDNF, AR, AMH, phos-Smad2/3, Smad2/3, Smad2/3, phos-Smad1/5/8, Smad1/5/8, phos-AKT, AKT, Phos-ERK1/2, ERK2, cyclin A, cyclin B1, cyclin D1, cyclin E, CDK2, and ACTB, and they were listed in Table S2. The membranes were incubated with horseradish peroxidase-conjugated anti-rabbit immunoglobulin G (IgG), anti-goat IgG, or anti-mouse IgG (Santa Cruz Biotechnology, CA, USA) at a 1:2000 dilution for 1 hour at room temperature. The blots were detected by chemiluminescence (Chemi-Doc XRS, Bio-Rad, Hercules, CA, USA), and densitometric analyses were processed with Adobe Photoshop 8.0. The relative levels of proteins, including phos-ERK2, phos-AKT, phos-Smad2/3, phos-Smad1/5/8, BMP6, ZO1, OCLN, SCF, GDNF, AR, AMH, cyclin A, cyclin B1, cyclin D1, cyclin E and CDK2, were normalized respectively to the expression of ERK2, AKT, Smad2/3, Smad1/5/8, and ACTB.

### RNA interference (RNAi) of BMP6, DACH1 and TFAP2A

The small interfering RNA (siRNA) sequences targeting human BMP6, DACH1, TFAP2A mRNA were purchased from GenePharma, Suzhou, China. The sequences of BMP6 siRNAs, DACH1 siRNAs, and TFAP2A siRNAs were listed in Tables S3,S4 and S5, respectively. To optimize the best effect of BMP6 RNAi, 4 siRNAs targeting different regions of *BMP6* were utilized in this study. Human Sertoli cells were seeded at 2 × 10^5^/cm^2^ density and cultured in DMEM/F-12 supplemented with 10% FBS (complete medium) for overnight to reach a confluence of about 80%. The medium was changed to DMEM/F-12 supplemented with 1% FBS. Transfection of the carboxyfluorescein (FAM)-labeled control siRNA, BMP6 siRNAs, DACH1 siRNAs, TFAP2A siRNAs using lipofectamine 2000 according to the manufacturer’s manual. In detail, 60 pmol of BMP6 siRNAs, DACH1 siRNAs, TFAP2A siRNAs, control siRNA, and the fluorescent oligo were diluted in 100 μl Opti-MEM I Reduced Serum Medium (Invitrogen) and mixed gently. Lipofectamine 2000 (2 μl) was diluted in 100 μl Opti-MEM I Reduced Serum Medium and incubated for 15 min at room temperature. After the 15-min incubation, the diluted siRNAs, control siRNA, or the fluorescent oligo were combined with the diluted Lipofectamine 2000 and incubated for another 15 min at room temperature to allow the oligomer-Lipofectamine 2000 complex formation. The culture medium was changed with 1 ml fresh DMEM/F-12 without antibiotics before transfection, and the oligomer-Lipofectamine 2000 complexes were added to each well and mixed gently. The medium with 1% FBS was changed to complete medium at 6 hours after transfection and the viability of the cells were evaluated by trypan blue staining. The transfection efficiency was detected using Nikon fluorescence microscope, and after 48 hours or 72 hours, the cells were harvested to examine the expression changes of various genes and proteins accordingly.

### Cell Counting Kit-8 (CCK-8) Proliferation Assay

Human Sertoli cells were seeded at a density of 2,000 cells/well in 96-well microtiter plates in DMEM/F-12 supplemented with 10% FBS overnight, and they were cultured with recombinant human BMP6 factor (Peprotech), or 0.1% BSA (control), or BMP6 siRNAs, DACH1 siRNAs, TFAP2A siRNAs and control siRNA in DMEM/F-12 supplemented with 1% FBS. The medium was changed every day. The proliferation potential of human Sertoli cells was detected by CCK-8 assay (Dojin Laboratories, Kumamoto, Japan) according to the manufacturer’s instruction.

### EDU Incorporation Assay

Human Sertoli cells were seeded in 96-well plates at 0.5 × 10^4^ cells/well in DMEM/F-12 supplemented with 10% FBS overnight to allow the cells to attach. Cells were starved in DMEM/F-12 for 16 hours, and they were exposed to 100 ng/ml recombinant human BMP6 factor, or 0.1% BSA (control), or control siRNA or BMP6 siRNA-1 for 18 hours in DMEM/F-12 supplemented with 1% FBS and 20 μΜ 5-Ethynyl-2′-Deoxyuridine (EDU) (RiboBio, Guangzhou, China). The cells were washed twice with PBS and fixed with 4% PFA at room temperature, and 50 μl of 2 mg/ml glycine (DingGuo ChangShengBiotech) was added to per well to neutralize the PFA. The cells were washed with 0.5% tritonx-100 in PBS and exposed with 100 μl Apollo-Fluor (RiboBio, Guangzhou, China) for 30 min in the dark at room temperature. Cells were stained with Hoechst 33342 for 30 min. The percentages of EDU-positive cells were counted from 500 cells and three independent experiments were performed.

### Annexin-V/Propidium Iodide (PI) Staining and Flow Cytometry

Human Sertoli cells seeded in 12-well plates (2.5 × 10^4^ cells/well) were exposed to 100 ng/ml recombinant human BMP6 factor or 0.1% BSA (control) for 72 hours in DMEM/F-12 supplemented with 1% FBS, or BMP6 siRNA-1, or control siRNA for 48 hours as previously described. Cells were harvested and washed with cold PBS, and apoptosis percentages of human Sertoli cells were detected using the Annexin V-FITC/PI kit by flow cytometry pursuant to the manufacturer’s instruction (Biolegend, London, UK). It was feasible to identify and quantify apoptotic cells by conjugating FITC to Annexin V using flow cytometry. Staining cells simultaneously with Annexin V-FITC (green fluorescence) and the non-vital dye PI (red fluorescence) allowed the discrimination of intact cells (FITC^−^PI^−^), early apoptotic (FITC^+^PI^−^) and late apoptotic or necrotic cells (FITC^+^PI^+^).

### Enzyme Linked Immunosorbent Assay (ELISA)

SCF synthesis of human Sertoli cells affected by BMP6 was evaluated by ELISA. Culture medium from human Sertoli cells cultured with recombinant human BMP6 or BMP6 siRNA-1 or control siRNA was collected every day and stored at −80 °C. The medium supernatant was concentrated with 10 kDa ultra filter units (Merk Millipore, Billerica, USA), and ELISA assay was performed according to the manufacturer’s instruction (R&D, Minneapolis, USA). The SCF secretion of the cells was normalized to per 10^6^ cells.

### Statistical Analysis

All data were presented as mean ± SEM from at least three independent experiments and analyzed by Student’s t-test or one way ANOVA with the appropriate post-hoc tests (Dunnet’s test for non-parametric data or Turkey’s multiple comparison for parametric data) using Prism (version 5, GraphPad Software). Normality and homogeneity of variances were checked prior to conduct Student’s t-test or one way ANOVA, and P < 0.05 was considered statistically significant.

## Additional Information

**How to cite this article**: Wang, H. *et al*. BMP6 Regulates Proliferation and Apoptosis of Human Sertoli Cells Via Smad2/3 and Cyclin D1 Pathway and DACH1 and TFAP2A Activation. *Sci. Rep.* **7**, 45298; doi: 10.1038/srep45298 (2017).

**Publisher's note:** Springer Nature remains neutral with regard to jurisdictional claims in published maps and institutional affiliations.

## Supplementary Material

Supplementary Information

## References

[b1] De Kretser D. M. & Baker H. W. Infertility in men: recent advances and continuing controversies. J Clin Endocrinol Metab 84, 3443–3450 (1999). 10522977 10.1210/jcem.84.10.6101

[b2] Wosnitzer M., Goldstein M. & Hardy M. P. Review of Azoospermia. Spermatogenesis 4, e28218 (2014). 25105055 10.4161/spmg.28218 PMC4124057

[b3] Hofmann M. C. Gdnf signaling pathways within the mammalian spermatogonial stem cell niche. Mol Cell Endocrinol 288, 95–103 (2008). 18485583 10.1016/j.mce.2008.04.012 PMC2491722

[b4] Orth J. M., Gunsalus G. L. & Lamperti A. A. Evidence from Sertoli cell-depleted rats indicates that spermatid number in adults depends on numbers of Sertoli cells produced during perinatal development. Endocrinology 122, 787–794 (1988). 3125042 10.1210/endo-122-3-787

[b5] Lavery K., Swain P., Falb D. & Alaoui-Ismaili M. H. BMP-2/4 and BMP-6/7 differentially utilize cell surface receptors to induce osteoblastic differentiation of human bone marrow-derived mesenchymal stem cells. J Biol Chem 283, 20948–20958 (2008). 18436533 10.1074/jbc.M800850200 PMC3258927

[b6] Mazerbourg S. & Hsueh A. J. Genomic analyses facilitate identification of receptors and signalling pathways for growth differentiation factor 9 and related orphan bone morphogenetic protein/growth differentiation factor ligands. Hum Reprod Update 12, 373–383 (2006). 16603567 10.1093/humupd/dml014

[b7] von Bubnoff A. & Cho K. W. Intracellular BMP signaling regulation in vertebrates: pathway or network? Dev Biol 239, 1–14 (2001). 11784015 10.1006/dbio.2001.0388

[b8] Hai Y. . BMP4 promotes human Sertoli cell proliferation via Smad1/5 and ID2/3 pathway and its abnormality is associated with azoospermia. Discovery medicine 19, 311–325 (2015). 25977194

[b9] Nicholls P. K., Harrison C. A., Gilchrist R. B., Farnworth P. G. & Stanton P. G. Growth differentiation factor 9 is a germ cell regulator of Sertoli cell function. Endocrinology 150, 2481–2490 (2009). 19106224 10.1210/en.2008-1048

[b10] Sekiya I., Colter D. C. & Prockop D. J. BMP-6 enhances chondrogenesis in a subpopulation of human marrow stromal cells. Biochem Biophys Res Commun 284, 411–418 (2001). 11394894 10.1006/bbrc.2001.4898

[b11] Song X. . Bmp signals from niche cells directly repress transcription of a differentiation-promoting gene, bag of marbles, in germline stem cells in the Drosophila ovary. Development 131, 1353–1364 (2004). 14973291 10.1242/dev.01026

[b12] Xie T. & Spradling A. C. decapentaplegic is essential for the maintenance and division of germline stem cells in the Drosophila ovary. Cell 94, 251–260 (1998). 9695953 10.1016/s0092-8674(00)81424-5

[b13] Chen D. & McKearin D. Dpp signaling silences bam transcription directly to establish asymmetric divisions of germline stem cells. Curr Biol 13, 1786–1791 (2003). 14561403 10.1016/j.cub.2003.09.033

[b14] Zhang J. . Identification of the haematopoietic stem cell niche and control of the niche size. Nature 425, 836–841 (2003). 14574412 10.1038/nature02041

[b15] Shiozawa Y. . Erythropoietin couples hematopoiesis with bone formation. PLoS One 5, e10853 (2010). 20523730 10.1371/journal.pone.0010853 PMC2877712

[b16] Peretto P. . BMP mRNA and protein expression in the developing mouse olfactory system. J Comp Neurol 451, 267–278 (2002). 12210138 10.1002/cne.10343

[b17] Crews L. . Increased BMP6 levels in the brains of Alzheimer’s disease patients and APP transgenic mice are accompanied by impaired neurogenesis. J Neurosci 30, 12252–12262 (2010). 20844121 10.1523/JNEUROSCI.1305-10.2010 PMC2978735

[b18] Massague J., Blain S. W. & Lo R. S. TGFbeta signaling in growth control, cancer, and heritable disorders. Cell 103, 295–309 (2000). 11057902 10.1016/s0092-8674(00)00121-5

[b19] Asanbaeva A., Masuda K., Thonar E. J., Klisch S. M. & Sah R. L. Regulation of immature cartilage growth by IGF-I, TGF-beta1, BMP-7, and PDGF-AB: role of metabolic balance between fixed charge and collagen network. Biomech Model Mechanobiol 7, 263–276 (2008). 17762943 10.1007/s10237-007-0096-8 PMC2704288

[b20] Tobin J. F. & Celeste A. J. Bone morphogenetic proteins and growth differentiation factors as drug targets in cardiovascular and metabolic disease. Drug Discov Today 11, 405–411 (2006). 16635802 10.1016/j.drudis.2006.03.016

[b21] Zhang Y. . Analysis and characterization of the functional TGFbeta receptors required for BMP6-induced osteogenic differentiation of mesenchymal progenitor cells. BMB Rep 46, 107–112 (2013). 23433114 10.5483/BMBRep.2013.46.2.141 PMC4133849

[b22] Gonen N., Quinn A., O’Neill H. C., Koopman P. & Lovell-Badge R. Normal Levels of Sox9 Expression in the Developing Mouse Testis Depend on the TES/TESCO Enhancer, but This Does Not Act Alone. PLoS Genet 13, e1006520 (2017). 28045957 10.1371/journal.pgen.1006520 PMC5207396

[b23] Lui W. Y., Mruk D., Lee W. M. & Cheng C. Y. Sertoli cell tight junction dynamics: their regulation during spermatogenesis. Biology of reproduction 68, 1087–1097 (2003). 12606453 10.1095/biolreprod.102.010371

[b24] Wu K. . DACH1 inhibits transforming growth factor-beta signaling through binding Smad4. J Biol Chem 278, 51673–51684 (2003). 14525983 10.1074/jbc.M310021200

[b25] Koinuma D. . Chromatin immunoprecipitation on microarray analysis of Smad2/3 binding sites reveals roles of ETS1 and TFAP2A in transforming growth factor beta signaling. Molecular and cellular biology 29, 172–186 (2009). 18955504 10.1128/MCB.01038-08 PMC2612478

[b26] Wang H. . Establishment and applications of male germ cell and Sertoli cell lines. Reproduction 152, R31–40 (2016). 27069011 10.1530/REP-15-0546

[b27] Sharpe R. M., McKinnell C., Kivlin C. & Fisher J. S. Proliferation and functional maturation of Sertoli cells, and their relevance to disorders of testis function in adulthood. Reproduction 125, 769–784 (2003). 12773099 10.1530/rep.0.1250769

[b28] Tarulli G. A., Stanton P. G. & Meachem S. J. Is the adult Sertoli cell terminally differentiated? Biol Reprod 87, 13, 11–11 (2012). 22492971 10.1095/biolreprod.111.095091

[b29] Tarulli G. A., Stanton P. G., Lerchl A. & Meachem S. J. Adult sertoli cells are not terminally differentiated in the Djungarian hamster: effect of FSH on proliferation and junction protein organization. Biol Reprod 74, 798–806 (2006). 16407497 10.1095/biolreprod.105.050450

[b30] Guo Y. . Long-term culture and significant expansion of human Sertoli cells whilst maintaining stable global phenotype and AKT and SMAD1/5 activation. Cell communication and signaling: CCS 13, 20 (2015). 25880873 10.1186/s12964-015-0101-2 PMC4380114

[b31] Schmidt J. A., Avarbock M. R., Tobias J. W., & Brinster R. L. Identification of glial cell line-derived neurotrophic factor-regulated genes important for spermatogonial stem cell self-renewal in the rat. Biol Reprod 81, 56–66 (2009). 19339709 10.1095/biolreprod.108.075358 PMC3093986

[b32] Oatley J. M., Avarbock M. R. & Brinster R. L. Glial cell line-derived neurotrophic factor regulation of genes essential for self-renewal of mouse spermatogonial stem cells is dependent on Src family kinase signaling. J Biol Chem 282, 25842–25851 (2007). 17597063 10.1074/jbc.M703474200 PMC4083858

[b33] He Z. . Gdnf upregulates c-Fos transcription via the Ras/Erk1/2 pathway to promote mouse spermatogonial stem cell proliferation. Stem Cells 26, 266–278 (2008). 17962702 10.1634/stemcells.2007-0436 PMC2905627

[b34] Kadam P. H. . Effects of glial cell line-derived neurotrophic factor, fibroblast growth factor 2 and epidermal growth factor on proliferation and the expression of some genes in buffalo (Bubalus bubalis) spermatogonial cells. Reproduction, fertility, and development 25, 1149–1157 (2013). 10.1071/RD12330 23171731

[b35] Mauduit C., Hamamah S. & Benahmed M. Stem cell factor/c-kit system in spermatogenesis. Hum Reprod Update 5, 535–545 (1999). 10582791 10.1093/humupd/5.5.535

[b36] Feng L. X., Ravindranath N. & Dym M. Stem cell factor/c-kit up-regulates cyclin D3 and promotes cell cycle progression via the phosphoinositide 3-kinase/p70 S6 kinase pathway in spermatogonia. J Biol Chem 275, 25572–25576 (2000). 10849422 10.1074/jbc.M002218200

[b37] Wang R. S., Yeh S., Tzeng C. R. & Chang C. Androgen receptor roles in spermatogenesis and fertility: lessons from testicular cell-specific androgen receptor knockout mice. Endocrine reviews 30, 119–132 (2009). 19176467 10.1210/er.2008-0025 PMC2662628

[b38] De Gendt K. . A Sertoli cell-selective knockout of the androgen receptor causes spermatogenic arrest in meiosis. Proceedings of the National Academy of Sciences of the United States of America 101, 1327–1332 (2004). 14745012 10.1073/pnas.0308114100 PMC337052

[b39] Chang C. . Infertility with defective spermatogenesis and hypotestosteronemia in male mice lacking the androgen receptor in Sertoli cells. Proceedings of the National Academy of Sciences of the United States of America 101, 6876–6881 (2004). 15107499 10.1073/pnas.0307306101 PMC406435

[b40] Tan K. A. . The role of androgens in sertoli cell proliferation and functional maturation: studies in mice with total or Sertoli cell-selective ablation of the androgen receptor. Endocrinology 146, 2674–2683 (2005). 15761038 10.1210/en.2004-1630

[b41] Tsai M. Y. . Differential effects of spermatogenesis and fertility in mice lacking androgen receptor in individual testis cells. Proceedings of the National Academy of Sciences of the United States of America 103, 18975–18980 (2006). 17142319 10.1073/pnas.0608565103 PMC1748162

[b42] Willems A. . Selective ablation of the androgen receptor in mouse sertoli cells affects sertoli cell maturation, barrier formation and cytoskeletal development. PLoS One 5, e14168 (2010). 21152390 10.1371/journal.pone.0014168 PMC2994754

[b43] Wang G. . Androgen receptor in Sertoli cells is not required for testosterone-induced suppression of spermatogenesis, but contributes to Sertoli cell organization in Utp14b jsd mice. Journal of andrology 30, 338–348 (2009). 19136388 10.2164/jandrol.108.006890 PMC2797546

[b44] Derynck R. & Zhang Y. E. Smad-dependent and Smad-independent pathways in TGF-beta family signalling. Nature 425, 577–584 (2003). 14534577 10.1038/nature02006

[b45] Yan W. . Epigenetic silencing of DACH1 induces the invasion and metastasis of gastric cancer by activating TGF-beta signalling. Journal of cellular and molecular medicine 18, 2499–2511 (2014). 24912879 10.1111/jcmm.12325 PMC4302654

[b46] Mitchell P. J., Wang C. & Tjian R. Positive and negative regulation of transcription *in vitro*: enhancer-binding protein AP-2 is inhibited by SV40 T antigen. Cell 50, 847–861 (1987). 3040262 10.1016/0092-8674(87)90512-5

[b47] Imagawa M., Chiu R. & Karin M. Transcription factor AP-2 mediates induction by two different signal-transduction pathways: protein kinase C and cAMP. Cell 51, 251–260 (1987). 2822255 10.1016/0092-8674(87)90152-8

[b48] Zeng Y. X., Somasundaram K. & el-Deiry W. S. AP2 inhibits cancer cell growth and activates p21WAF1/CIP1 expression. Nature genetics 15, 78–82 (1997). 8988173 10.1038/ng0197-78

[b49] Liu Y. . Fractionation of human spermatogenic cells using STA-PUT gravity sedimentation and their miRNA profiling. Sci Rep 5, 8084 (2015). 25634318 10.1038/srep08084 PMC5155379

[b50] He Z., Jiang J., Hofmann M. C. & Dym M. Gfra1 silencing in mouse spermatogonial stem cells results in their differentiation via the inactivation of RET tyrosine kinase. Biology of reproduction 77, 723–733 (2007). 17625109 10.1095/biolreprod.107.062513 PMC2911237

[b51] Hadad N. . Absence of genomic hypomethylation or regulation of cytosine-modifying enzymes with aging in male and female mice. Epigenetics & chromatin 9, 30 (2016). 27413395 10.1186/s13072-016-0080-6 PMC4942942

[b52] Jones N. R. & Lazarus P. UGT2B gene expression analysis in multiple tobacco carcinogen-targeted tissues. Drug metabolism and disposition: the biological fate of chemicals 42, 529–536 (2014). 24459179 10.1124/dmd.113.054718 PMC3965906

[b53] Yang B. . Methylation of Dickkopf-3 as a prognostic factor in cirrhosis-related hepatocellular carcinoma. World journal of gastroenterology 16, 755–763 (2010). 20135726 10.3748/wjg.v16.i6.755 PMC2817066

[b54] Gonzalez T. . Use of multiplex reverse transcription-PCR to study the expression of a laccase gene family in a basidiomycetous fungus. Applied and environmental microbiology 69, 7083–7090 (2003). 14660352 10.1128/AEM.69.12.7083-7090.2003 PMC310016

