# Supplementary material for: BMP6 Regulates Proliferation and Apoptosis of Human Sertoli Cells Via Smad2/3 and Cyclin D1 Pathway and DACH1 and TFAP2A Activation
Source: Sci Rep. 2017 Apr 7;7:45298. doi: 10.1038/srep45298 (PMC5384448; doi:10.1038/srep45298)
Supplement: Supplementary Information [file srep45298-s1.pdf]

BMP6 Regulates Proliferation and Apoptosis of Human Sertoli Cells Via Smad2/3 and Cyclin D1 Pathway and DACH1 and TFAP2A Activation

Hong Wang, Qingqing Yuan, Min Sun, Minghui Niu, Liping Wen, Hongyong Fu, Fan Zhou, Zheng Chen, Chencheng Yao, Jingmei Hou, Ruinan Shen, Qisheng Lin, Wenjie Liu, Ruobing Jia, Zheng Li & Zuping He

Supplementary Figure

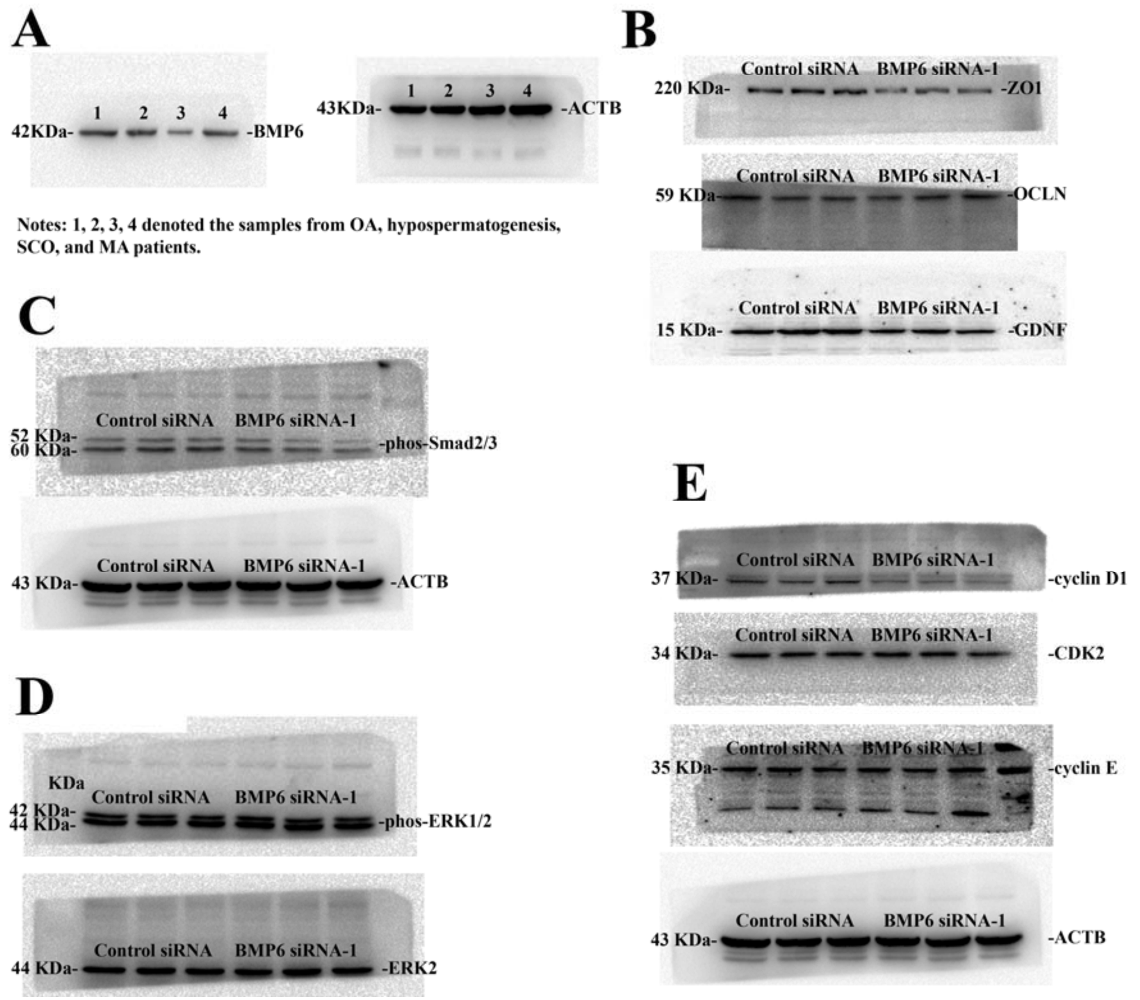

Notes: 1, 2, 3, 4 denoted the samples from OA, hypospermatogenesis, SCO, and MA patients.

Supplementary Tables

Table S1. Primer sequences used for RT-PCR and quantitative real-time PCR.

Genes	Primer sequences	Product size (bp)	Tm (℃)	Efficiency
GATA4	F: 5'-GCCTCCTCTGCCTGGTAAT-3'<br>R: 5'-CAGTCCCATCAGCGTGTAAA-3'	120	60	—
GATA1	F: 5'-GAAACCGCAAGGCATCTG-3'<br>R: 5'-CCCAGCCACCACCATAAAG-3'	140	60	—
GDNF	F: 5'-CGGAGGAGGAGGAAGAAGAA-3'<br>R: 5'-GGAGAATCCAGAGGGCTGTT-3'	142	60	—
FGF2	F: 5'-TCCTTTCTCCCTCGTTTCTTC-3'<br>R: 5'-GATGTTTCCCTCCAATGTTTC-3'	144	60	—
BMP4	F: 5'-TTTGTTCAAGATTGGCTGTC-3'<br>R: 5'-AGATCCCGCATGTAGTCC-3'	324	57	—
AR	F: 5'-AAAGCCTACCCAAGTGATTGA-3'<br>R: 5'-AGGAAACTGCCCTTCTCTCA-3'	110	60	—
SCF	F: 5'-GTCATTGTTGGATAAGCGAGAT-3'<br>R: 5'-ATGGCTGCCCAGTGTAGG-3'	457	55	—
WT1	F:5'-TGACTCTCCACTCCTCCTCAC-3'<br>R: 5'-ACCAACTCTTCCAGGCACAC-3'	115	60	—
SOX9	F:5'-AGGTGCTCAAAGGCTACGACTG-3'<br>R: 5'-TGCCCGTTCTTCACCGACT-3'	322	60	—
BMP2	Quantitative real time PCR:<br>F:5'-ACCCGCTGTCTTCTAGCGT-3'<br>R: 5'-TTTCAGGCCGAACATGCTGAG-3'	180	60	105% R^2=0.999
BMP4	Quantitative real time PCR:<br>F:5'-AAAGTCGCCGAGATTCAGGG-3'<br>R: 5'-GACGGCACTCTTGCTAGGC-3'	135	60	98% R^2=0.985
BMP9	Quantitative real time PCR:<br>F:5'-AGAACGTGAAGGTGGATTTCC-3'<br>R: 5'-CGCACAATGTTGGACGCTG-3'	151	60	103% R^2=0.991
BMP15	Quantitative real time PCR:<br>F:5'-AGACCAAACCGAGGACTATACC-3'<br>R: 5'-GAGATTGAAGCGAGTTAGTTGGA-3'	81	59	105% R^2=0.997
BMP6	RT-PCR and Quantitative real time PCR:<br>F:5'-AGCGACACCACAAAGAGTTCA-3'<br>R: 5'- GCTGATGCTCCTGTAAGACTTGA-3'	159	60	94% R^2=0.997
ACVR2A	F:5'-CAACATCCTGCTCTATTCC-3'<br>R: 5'-CATCAACACTGGTGCCTC-3'	371	55	—
ACVR2B	F:5'-CGGACCCGTGGATGAGTA-3'<br>R: 5'-CCGAGGTAGTGCCGTTGA-3'	260	58	—
ACVR1	F:5'-AGCATTGGTAAGCGTCAC-3'<br>R: 5'-TCCCTGCTCATAAACCTG-3'	408	54	—

BMPR1A	F: 5'-AAGTTCTGGTAGTGGGTCT-3'	168	50	–
	R: 5'-CTGGCTTCTTCAGTGGTA-3'			
BMPR1B	F: 5'-ACATACCACCTAACACTCG-3'	403	52	–
	R: 5'-TCCTGGGACTCTGACATT-3'			
BMPR2	F: 5'-TCAAGAACGGCTATGTGCGT-3'	595	60	–
	R: 5'-AACTGGACGCTCATCCAAGG-3'			
DACH1	Quantitative real time PCR:	76	60	92% R^2=0.999
	F:5'-CGAACTTGTTCCACATTGCACA-3'			
	R: 5'-GGGGCTTGCATACGGTCTAC-3'			
TFAP2A	Quantitative real time PCR:	121	60	108% R^2=0.992
	F:5'-GGAGTAAGGATCTTGCGACTGG-3'			
	R: 5'-AGGTCAATCTCCCTACACGAG-3'			
GAPDH	Quantitative real time PCR:	197	60	95% R^2=0.989
	F:5'-GGAGCGAGATCCCTCCAAAAT-3'			
	R: 5'-GGCTGTTGTCATACTTCTCATGG-3'			
VASA	F: 5'-GCAGAAGGAGGAGAAAGTAGTGG-3'	289	60	
	R: 5'-CTCGTCCTGCAAGTATGATAGG-3'			
ACTB	F:5'-CGCACCACTGGCATTGTCAT-3'	253	55	–
	R: 5'- TTCTCCTTGATGTCACGCAC-3'			
ACTB	RT-PCR and Quantitative real time PCR:	144	60	94% R^2=0.990
	F:5'-GGGCCGGACTCGTCATAC-3'			
	R: 5'- CCTGGCACCCAGCACAAT-3'			

Table S2. Primary antibodies used for Western blots, immunohistochemistry, and immunocytochemistry.

**Antibodies**	**Sources**	**Vendors**	**Working dilutions**
WT1	Rabbit	Santa Cruz	ICC: 1:200
BMP4	Rabbit	Abcam	ICC: 1:100
SOX9	Rabbit	Millipore	IHC: 1:500; ICC: 1:500
OCLN	Rabbit	Abcam	WB: 1:500; ICC: 1:200
ZO1	Rabbit	Cell Signaling	WB: 1:500; ICC: 1:200
SCF	Rabbit	Santa Cruz	WB: 1:300
VASA	goat	Santa Cruz	ICC:1:100
AMH	Mouse	Santa Cruz	WB: 1:200
GDNF	Rabbit	Santa Cruz	WB: 1:300
BMP6	Rabbit	Abcam	WB: 1:500;IHC: 1:200; ICC: 1:200
ACVR1	Rabbit	Abcam	WB: 1:500
BMPR1A	Rabbit	Abcam	WB: 1:1000; IHC: 1:200; ICC: 1:50
BMPR1B	Rabbit	Santa Cruz	WB: 1:100; ICC: 1:50
BMPR2	Goat	Santa Cruz	WB: 1:500; IHC: 1:100; ICC: 1:50
Phos-Smad2/3	Rabbit	Cell Signaling	WB: 1:500
Smad2/3	Rabbit	Cell Signaling	WB: 1:500
Phos-Smad1/5/8	Rabbit	Cell Signaling	WB: 1:500
Smad1/5/8	Rabbit	Cell Signaling	WB: 1:1000
Phos-ERK1/2	Rabbit	Cell Signaling	WB: 1:1000
ERK2	Mouse	Santa Cruz	WB: 1:100
Phos-AKT	Rabbit	Abcam	WB: 1:500
AKT	Rabbit	Abcam	WB: 1:500
Cyclin A	Rabbit	Santa Cruz	WB: 1:100
Cyclin B1	Rabbit	Santa Cruz	WB: 1:100
Cyclin D1	Rabbit	Santa Cruz	WB: 1:100
Cyclin E	Mouse	Santa Cruz	WB: 1:100
ACTB	Mouse	Proteintech	WB: 1:5000
GAPDH	Mouse	Yeasen	WB:1:2000

Table S3. The sequences of oligonucleotides for human BMP6 siRNAs

siRNA	siRNA sequences(5'-3')		Knockdown rate
BMP6 siRNA-1	Sense	GCAGACCUUGGUUCACCUUTT	88.2%±2.4%
	Antisense	AAGGUGAACCAAGGUCUGCTT	
BMP6 siRNA-2	Sense	CCUUCAUGGUGGCUUUCUUTT	81.3%±1.8%
	Antisense	AAGAAAGCCACCAUGAAGGTT	
BMP6 siRNA-3	Antisense	CCGACAACAGAGUCGUAAUTT	81.5%±4.6%
	Antisense	AUUACGACUCUGUUGUCGGTT	
BMP6 siRNA-4	Sense	GGUUGUGACUCCACAGCAUTT	64.6%±3.6%
	Antisense	AUGCUGUGGAGUCACAACCTT	
Negative control	Sense	UUCUCCGAACGUGUCACGUTT	
	Antisense	ACGUGACACGUUCGGAGAATT	
Negative control FAM	Sense	UUCUCCGAACGUGUCACGUTT	
	Antisense	ACGUGACACGUUCGGAGAATT	

Table S4. The sequences of oligonucleotides for human DACH1 siRNAs

siRNA	siRNA sequences(5'-3')		Knockdown rate
DACH1 siRNA-1	Sense	GGGCUUAUCACCAAAUGUATT	72.1%±2.4%
	Antisense	UACAUUUGGUGAUAAGCCCTT	
DACH1 siRNA-2	Sense	CCUCCUAAGAGGACUCAAATT	31.8%±6.7%
	Antisense	UUUGAGUCCUCUUAGGAGGTT	
DACH1 siRNA-3	Sense	GCAGUUGGCUAUGGAACAATT	67.0%±3.7%
	Antisense	UUGUUCCAUAGCCAACUGCTT	
Negative control	Sense	UUCUCCGAACGUGUCACGUTT	
	Antisense	ACGUGACACGUUCGGAGAATT	

Table S5. The sequences of oligonucleotides for human TFAP2A siRNAs

siRNA	siRNA sequences(5'-3')		Knockdown rate
TFAP2A siRNA-1	Sense	CCAGAUCAAACUGUAAUUATT	53.8%±2.2%
	Antisense	UAAUUACAGUUUGAUCUGGTT	
TFAP2A siRNA-2	Sense	CCUGCUCACAUCACUAGUATT	51.2%±2.2%
	Antisense	UACUAGUGAUGUGAGCAGGTT	
TFAP2A siRNA-3	Sense	GGAAGAUCUUUAAGAGAAATT	34.7%±4.4%
	Antisense	UUUCUCUUAAAGAUCUUCCTT	
Negative control	Sense	UUCUCCGAACGUGUCACGUTT	
	Antisense	ACGUGACACGUUCGGAGAATT	
